# Effect of linalool on the acquisition and reinstatement of morphine-induced conditioned place preference in mice

**Published:** 2017

**Authors:** Narjes Pourtaqi, Mohsen Imenshahidi, Bibi Marjan Razavi, Hossein Hosseinzadeh

**Affiliations:** 1 *School of Pharmacy, Mashhad University of Medical Sciences, Mashhad, Iran*; 2 *Pharmaceutical Research Center, Department of Pharmacodynamics and Toxicology, School of Pharmacy, Mashhad University of Medical Sciences, Mashhad, Iran*; 3 *Targeted Drug Delivery Research Center, Department of Pharmacodynamics and Toxicology, School of Pharmacy, Mashhad University of Medical Sciences, Mashhad, Iran*

**Keywords:** Conditioned place preference (CPP), Linalool, Memantine, Mice, Morphine

## Abstract

**Objective:**

The effect of linalool, a terpene alcohol found in many plants, which inhibits NMDA receptors, on the acquisition and reinstatement of morphine-induced conditioned place preference (CPP) was evaluated in mice.

**Material and Methods:**

The effects of different doses of linalool (12.5, 25 and 50 mg/kg, i.p.), memantine (20 mg/kg, an NMDA receptor antagonist) and saline, in CPP induced by 40 mg/kg of morphine were investigated in mice. In another experiment, a single injection of morphine (10 mg/kg) reinstated the place reference following extinction of a place preference induced by morphine (40 mg/kg). Linalool (12.5, 25 and 50 mg/kg, i.p.), memantine (20 mg/kg) and saline were administrated 30 min before this priming dose of morphine.

**Results:**

In the first experiment, linalool (12.5 and 50 mg/kg) was able to decrease morphine-induced CPP. In the second part, linalool (25 and 50 mg/kg) reduced morphine-induced reinstatement of place preference. Both acquisition and reinstatement of morphine-induced CPP, were considerably decreased by memantine.

**Conclusion:**

The present study showed that linalool is able to reduce the acquisition and reinstatement of morphine-induced CPP which might be due tothrough NMDA receptors blocking.

## Introduction

Opioid addiction is a kind of complex brain disorder, which is characterized by uncontrollable opioid craving behavior regardless of consequences. The high incidence of drug craving relapse following periods of abstinence is the main problem for treatment of drug abuse (O’Brien, 1997 ▶).

Increasing synaptic dopamine levels in the mesolimbic dopamine system, including the ventral tegmental area (VTA) and nucleus accumbens are responsible for rewarding effects of addictive drugs (Dong et al., 2006 ▶). Functional and morphological changes in mesolimbic dopamine system can be induced by chronic administration of morphine (Chu et al., 2007 ▶). Repeated morphine injection in a distinct environment and normal saline in another environment, forced animals to spend more time in the morphine-paired environment for drug craving. This process, known as the conditioned place preference (CPP), is generally used for discovering the effects rewarding of drugs (Bardo and Bevins, 2000 ▶; Cami and Farre, 2003 ▶).

Moreover, mesolimbic dopamine system can be indirectly stimulated by morphine through inhibition of GABAergic interneurons in the VTA which in turn increases the dopamine transmission to the nucleus accumbens (Johnson et al., 1992 ▶). In addition to this dopaminergic pathway, biochemical studies have shown that dopamine release can be regulated by glutamate and NMDA receptors. Opiate reward can also be modulated by NMDA receptors and the glutamatergic system. Therefore, glutamatergic system plays an important role in the development of morphine-induced CPP (Tzschentke and Schmidt, 1995 ▶). It has been shown that memantine, an NMDA receptor antagonist is able to prevent the acquisition of morphine-induced CPP (Do Couto et al., 2004 ▶). 

Other mechanisms including oxidative/nitrosative stress and induction of apoptosis in hypothalamus and hippocampus are also involved in morphine-induced CPP (Do Couto et al., 2005 ▶). 

Linalool is a naturally occurring monoterpene alcohol with a pleasing odor (Lee et al., 2007 ▶), which is found in oil and fruits of different plants, such as citrus, basil, coriander, lavender, etc. (Fisher and Phillips, 2008 ▶). Linalool is among the most common fragrance constituents in some products such as cosmetics and household products (Rastogi et al., 1998 ▶; Buckley, 2007 ▶).

A good bioactivity of linalool against different microorganisms has been reported (Kotan et al., 2007 ▶; Krist et al., 2008 ▶). Furthermore, several studies showed beneficial properties of this terpene including anti-inflammatory effects (Huo et al., 2013 ▶), local anesthetic (Ghelardini et al., 1999 ▶) and antioxidant activities (Celik and Ozkaya, 2002 ▶). Moreover, linalool possesses analgesic effect mediated through central nervous system (CNS) and peripheral mechanisms (Dong et al., 2006 ▶; Venâncio et al., 2011 ▶). Linalool can also inhibit glutamate activation *in vitro* and *in vivo* through competitive antagonism of NMDA ionotropic receptors (Elisabetsky et al., 1995 ▶; Brum et al., 2001 ▶; Elisabetsky and Silva Brum, 2003). The glutamatergic system is also involved in the anti-nociceptive activity of linalool through NMDA, AMPA and kainate ionotropic receptors (Dong et al., 2006 ▶).

It was indicated some plants (Tabatabai et al., 2014 ▶) such as *Crocus sativus* L. (Hosseinzadeh and Jahanian, 2009 ▶; Imenshahidi et al., 2011 ▶), *Berberis vulgaris* (Nassiri-Asl et al., 2007 ▶; Imenshahidi et al., 2014 ▶) and *Rosmarinus officinalis* L. (Hosseinzadeh and Nourbakhsh, 2003 ▶) have an important effect on morphine tolerance and dependence. Linalool also showed effects on morphine tolerance and dependence. This effect may be partly mediated through the inhibition of NMDA receptors (Elisabetsky et al., 1995 ▶; Hosseinzadeh et al., 2012 ▶). 

Linalool is believed to be an NMDA receptor blocker, and plays an important role in the reduction of morphine tolerance and dependence. So, in this study, the effect of linalool on the acquisition and reinstatement of morphine-induced CPP in mice was investigated.

## Materials and Methods

**Animals**

All experiments were performed in male mice, weighing 20-25g, that were housed four per cage under a 12 h/ 12 h light/dark cycle in a room with controlled temperature (25°C). Food and water were available *ad libitum*. All animals were administered in accordance with the guidelines for the care and use of laboratory animals prepared by the Animal Care Committee of Mashhad University of Medical Sciences. Ethical Committee Acts (Number of verification: 910451; the date of approval: 31.10.2012).

**Materials**

Linalool was purchased from Sigma-Aldrich (Sigma-Aldrich, Germany, liquid, purity 97%), memantine (Osve-Iran, powder), and morphine sulfate (DaruPakhsh-Iran) were also provided. Normal saline (NaCl 0.9%), was used as a solvent. 

**CPP apparatus**

The Plexiglas boxes had three compartments. Two chambers of the same size (30 cm length × 30 cm width × 35 cm height) were connected by the grey third chamber (15 cm length × 30 cm width × 35 cm height). The chambers walls had different colors (black vs. white) with similar floor textures (fine and wide grid in the black and white compartment, respectively). In order to provide the olfactory difference between the compartments, a drop of banana extract and acetic acid were placed at the corner of the black and white compartment floors, respectively. Moreover, whole box were cleaned for each test, to prevent the interference of odor produced by feces and/or urine.

**Experimental procedure**

**Acquisition of place preference**

**Pre-Conditioning Phase**

The study had three phases. The first phase lasted for three days. In the first two days, animals were permitted to move freely in each chamber for 20 min. On the third day, the time spent by the animal in each compartment was recorded for 15 min. The animals who had strong unconditioned preference (more than 66% of the session time) for any compartment were excluded (Do Couto et al., 2004 ▶).

**Conditioning Phase**

This phase lasted for four days. Animals were treated with a single i.p. administration of normal saline before being confined to the black compartment for 1 hr. After 4 hr, animals were treated with drugs (i.p.) immediately before confinement in the white compartment for 1 hr. Animals were divided into seven groups (n = 7):

1) saline. 2) morphine 40 mg/kg. 3) morphine 40 mg/kg + memantine 20 mg/kg. 4, 5, and 6) morphine 40 mg/kg + linalool 12.5, 25 and 50 mg/kg.

7) linalool 50 mg/kg (Do Couto et al., 2004 ▶; Hosseinzadeh et al., 2012 ▶).

**Post-conditioning phase**

On the 8th day of the study, guillotine doors were removed. The total time spent by each animal in each compartment was recorded for 15 min. The time spent in the central area was proportionally divided between both conditioning compartments. The difference of time spent by each mouse in the white compartment during pre and post-conditioning phases was calculated. If the result is a positive number, it means that the drug has induced a preference and vice versa (Do Couto et al., 2004 ▶).

**Extinction of place preference**

In this step the effects of linalool on morphine-induced CPP reinforcement were evaluated. Three phases of pre-conditioning, conditioning and post-conditioning were performed as described before for saline + morphine (40 mg/kg) group, then, animals were placed into the CPP chamber for 60 min daily for seven days (without partition separating the compartments) without any drug administration (Do Couto et al., 2005 ▶).

**Reinstatement of place preference**

On the 16th day of the study, the total time spent by each animal in each compartment was recorded for 15 min before administration of a single dose of morphine 10 mg /kg. Time spent in the white compartment for each group of animals was similar to those of pre-conditioning phase. Then, inducing the reinstatement by CPP was performed through injection of reminding 10 mg/kg morphine. Thirty minutes before the morphine priming dose, linalool (12.5, 25, 50 mg/kg) or normal saline were administrated. After 30 min, each animal was placed into the CPP chambers for 15 min similar to post-conditioning phase and the time spent in black and white chambers, was calculated (Do Couto et al., 2005 ▶).

**Statistical analysis**

Data were presented as mean ± SEM. Results were analyzed using two way ANOVA-repeated measures. The statistical significance was defined as p<0.05.

## Results

**Effects of linalool on acquisition of morphine-induced CPP**

Two way ANOVA-repeated measures [treatment effect: F (1, 6) = 129. 1, P<0.0001, dose effect (6, 36) = 1.484, P = 0.2113, treatment × dose interaction: F (6, 36) = 3.365, P=0.0062] indicated that pretreatment with linalool attenuated the acquisition of morphine-induced CPP.

**Figure 1:**
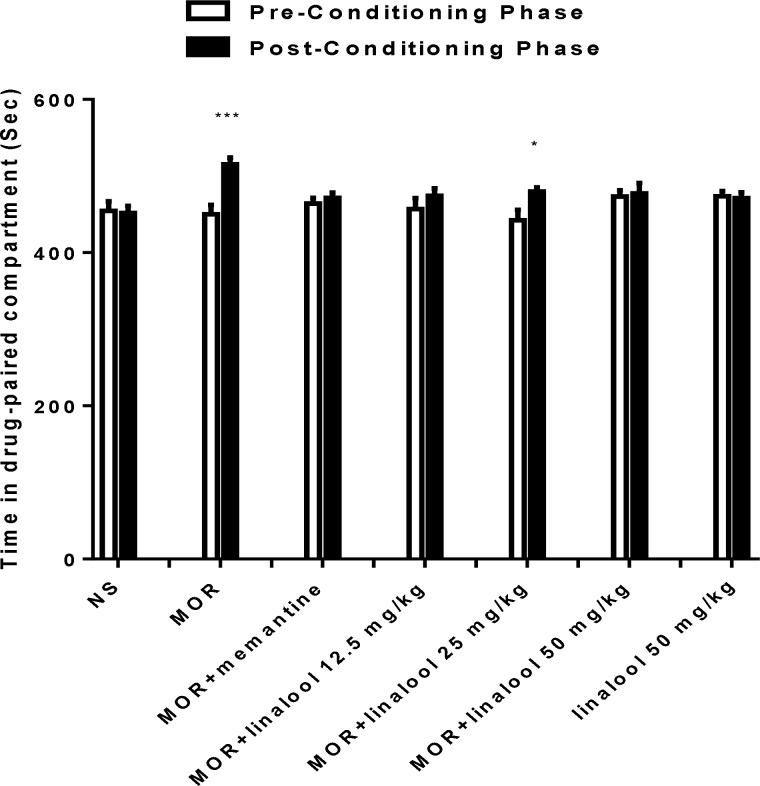
Effects of linalool (12.5, 25 and 50 mg/kg) on the acquisition of morphine-induced CPP in mice. During the conditioning phase animals received different treatments in the drug-paired compartment. Data are expressed as mean ± SEM of 7 animals per group. The bars represent the time spent in the drug-paired compartment before conditioning sessions in pre-conditioning test and after conditioning sessions in post-conditioning test. ^*^p<0.05 and ^***^p<0.001 significant differences in the time spent in the drug-paired compartment in pre-conditioning vs. post-conditioning sessions tests, NS: normal saline; Mor: Morphine

According to Figure 1, animals in morphine group acquired CPP after repeated administration of morphine (p<0.001). There was no significant difference between the time spent in pre and post-conditioning phases in linalool (12.5 and 50 mg/kg)-treated mice. It means that the administration of linalool during conditioning phase can prevent acquisition of morphine-induced CPP.

In memantine group (20 mg/kg), there was no significant difference between the time spent in pre- and post-conditioning phases. It means that memantine prevents acquisition of morphine-induced CPP during conditioning phase. The administration of linalool (50 mg/kg) could not induce CPP (Figure 1).

**Effects of linalool on reinstatement of morphine-induced CPP**

**Figure 2:**
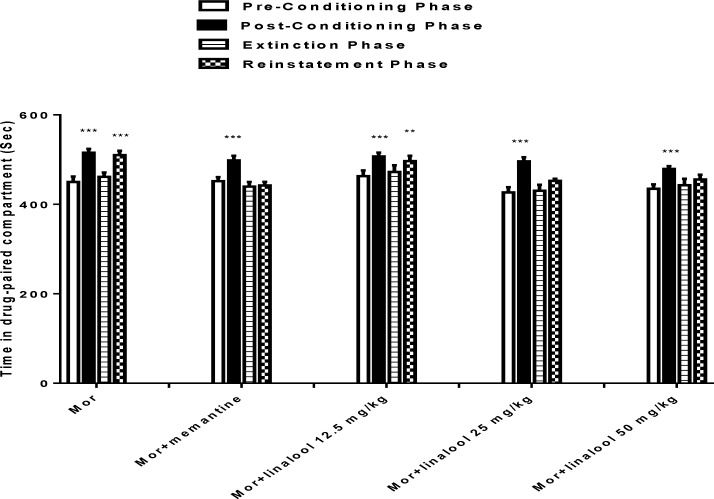
Effects of linalool (12.5, 25 and 50 mg/kg) on the reinstatement of morphine-induced CPP. Data are expressed as mean ± SEM of 7 animals per group. The bars represent the time spent in the drug-paired compartment before conditioning sessions (white bars), after conditioning sessions (black bars), in the last extinction session and in the reinstatement test. ^**^ p<0.01 and ^***^p<0.001 significant difference in the time spent in pre-conditioning vs. post-conditioning sessions or reinstatement tests. Mor: Morphine

As shown in Figure 2, there was no significant difference between pre-conditioning and the extinction session of each group. Following administration of morphine 10 mg/kg, the animals spent significantly more time in the drug-paired compartment during reinstatement tests in comparison with pre-conditioning (p<0.001). Repeated measure ANOVA, [treatment effect: F (3, 18) = 63.88 P<0.0001, dose effect (4, 24) = 4.614, P=0.0066, treatment × dose interaction: F (12,72) =2.973, P=0.002] indicated that pretreatment with linalool attenuated the morphine reinstatement. As shown in Figure 2, administration of linalool (12.5 mg/kg) could not inhibit morphine reinstatement (p<0.01 vs pre-conditioning) while linalool (25 and 50 mg/kg) significantly inhibited reinstatement of morphine. Also, memantine (10 mg/kg) inhibited morphine reinstatement in the current study.

**Comparing the effect of different linalool doses on reinstatement of place preference**

**Figure 3:**
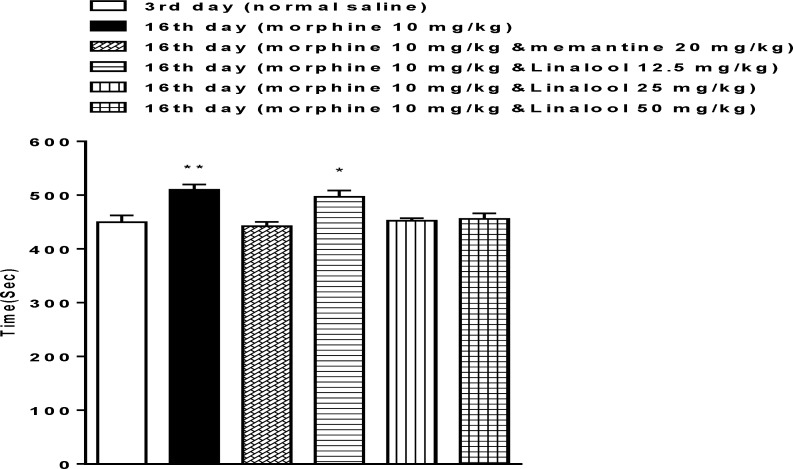
Comparing the effects of different linalool doses on the reinstatement of morphine-induced conditioned place preferences. Animals received morphine 40 mg/kg for 4 consecutive days and on the 16th day, saline or different doses of linalool (12.5, 25 and 50 mg/kg) or memantine (20 mg/kg), and morphine (10 mg/kg) were injected. 3rd day, pre-conditioning phase, and 16th day, reinstatement phase. The bars represent the mean ± S.E.M. time spent in the drug paired (white) in 7 animals. ^*^ p<0.05 and ^**^ p<0.01 significant difference in the time spent in pre-conditioning sessions vs. reinstatement tests

According to Figure 3, there were no significant differences between the 3rd and 1the 6th day after the single dose of morphine in linalool (25 and 50 mg/kg) and memantine groups. Thus, memantine and linalool inhibited the reinstatement of place preference because of priming dose of morphine (Figure 3).

## Discussion

Our results showed that linalool (12.5 and 50 mg/kg) could reduce acquisition of morphine-induced CPP. Furthermore, linalool (25 and 50 mg/kg) reduced morphine reinforcement that was induced by injection of a single reminding dose of morphine on the 16th day. Results also showed that linalool administration (50 mg/kg) did not induce CPP or aversion by itself. Because of linalool sedative effects at high doses (>100 mg/kg), lower doses (12.5, 25 and 50 mg/kg) were used in this study. It has been reported that these doses of linalool can not produce statistically significant effects in locomotor activity (Peana et al., 2003 ▶). CPP has been considered as one of the most popular methods to evaluate drug reward in laboratory animals. The pivotal roles of different neurotransmitter systems including dopaminergic, adrenergic, glutamatergic and GABAergic have been established in morphine-induced CPP. Besides the nucleus accumbens and ventral tegmental, different brain regions especially those which are involved in learning and memory process such as hippocampus, play an important role in CPP induction (Zarrindast et al., 2003 ▶). Studies indicated that activation of NMDA receptors in the nucleus accumbens and VTA is necessary for morphine's rewarding action; hence it is clear that NMDA receptor antagonists such as memantine could inhibit morphine’s rewarding properties in the CPP (Popik and Kolasiewicz, 1999 ▶; Siahposht-Khachaki et al., 2016 ▶). It has been shown that linalool inhibits NMDA receptors; therefore, the effect of linalool on the reduction of morphine-induced CPP may be partially related to the inhibition of NMDA receptors. According to several studies, NMDA antagonists could reduce acquisition and reinstatement of morphine-induced CPP. In this study, memantine was used as a positive control and results indicated that memantine reduced both acquisition and reinstatement of morphine-induced CPP. Previously, the major role of linalool in the reduction of tolerance and dependence induced by morphine has been shown to be probably due to several mechanisms including NMDA receptor inhibition, effect on NO signaling and adenosine receptor stimulation properties (Peana et al., 2004 ▶; Peana et al., 2006 ▶).

It was clarified that glutamate is a modulator of dopamine release. Dopamine has an important role in the rewarding pathway (Ribeiro et al., 2005 ▶). Dopamine-induced euphoria leads to morphine reinforcement. It has been established that infusion of NMDA into the nucleus accumbens led to the increase in dopamine level (Do Couto et al., 2004 ▶). Therefore, the role of glutamatergic pathway of the nucleus accumbens on the regulation of mesolimbic dopaminergic system as well as induction of opioid addiction has been established. Studies have indicated that dopamine antagonists can reduce morphine tendency in CPP model (Cami and Farre, 2003 ▶).

As a result, linalool could significantly reduce the acquisition and reinstatement of morphine-induced CPP. The effect of linalool on CPP may be due to its inhibitory effects on NMDA receptors. According to the results of this study, linalool may be used in drug addiction treatment.

## Conflict of interest statement

None of the authors have any conflict of interest.
